# Bis(1,2-dimethoxyethane)-1κ^2^ *O*,*O*′;3κ^2^ *O*,*O*′-tetra­kis­(μ-1,1,1,3,3,3-hexa­fluoro-2-methyl­propan-2-olato)-1:2κ^4^ *O*:*O*;2:3κ^4^ *O*:*O*-1,3-dilithium-2-magnesium

**DOI:** 10.1107/S1600536812032679

**Published:** 2012-07-25

**Authors:** Klaus Wurst, Michael R. Buchmeiser

**Affiliations:** a Institut für Allgemeine und Anorganische und Theoretische Chemie, Universität Innsbruck, Innrain 82, A-6020 Innsbruck, Austria; b Lehrstuhl für Makromolekulare Stoffe und Faserchemie, Institut für Polymerchemie, Universität Stuttgart, Pfaffenwaldring 55, D-70569 Stuttgart, Germany

## Abstract

The title compound, [Li_2_Mg(C_4_H_3_F_6_O)_4_(C_4_H_10_O_2_)_2_], forms as a white crystalline powder by-product of the reaction of lithium 1,1,1,3,3,3-hexa­fluoro-2-methyl-2-propoxide with Mo(N-2,6-Me_2_—C_6_H_3_)(CHCMe_2_Ph)(O_3_SCF_3_)_2_·2DME (DME is 1,2-dimethoxyethane) contaminated with MgCl_2_. The crystal structure of this compound contains half a mol­ecule in the asymmetric unit, with a twofold rotation axis through the central Mg^2+^ cation. The four 1,1,1,3,3,3-hexa­fluoro-2-methyl­propan-2-olate ligands serve as bridging ligands connecting the Li^+^ and Mg^2+^ cations. The Li^+^ cation is additionally stabilized by a DME ligand. This results in a distorted tetra­hedral ligand field around both the Mg^2+^ and Li^+^ cations.

## Related literature

For general background on the properties and synthesis of Schrock-type catalysts, see: Oskam *et al.* (1993 ▶). 
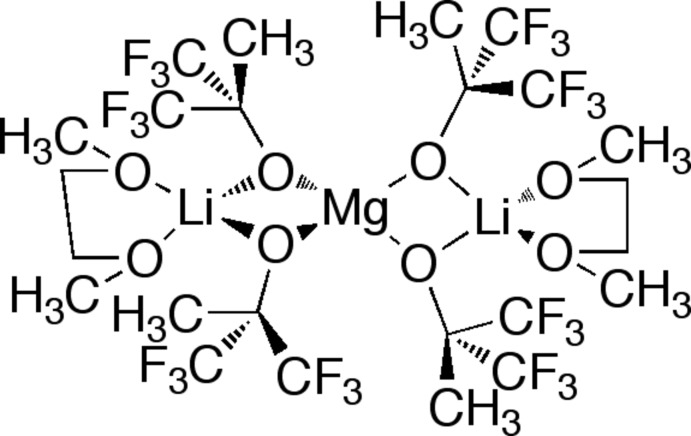


## Experimental

### 

#### Crystal data

[Li_2_Mg(C_4_H_3_F_6_O)_4_(C_4_H_10_O_2_)_2_]
*M* *_r_* = 942.69
Monoclinic, 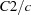

*a* = 23.8629 (4) Å
*b* = 9.5396 (6) Å
*c* = 18.3700 (7) Å
β = 109.041 (2)°
*V* = 3953.0 (3) Å^3^
*Z* = 4
Mo *K*α radiation
μ = 0.20 mm^−1^
*T* = 233 K
0.41 × 0.25 × 0.07 mm

#### Data collection

Nonius KappaCCD diffractometer
10600 measured reflections
3490 independent reflections
2603 reflections with *I* > 2σ(*I*)
*R*_int_ = 0.031

#### Refinement

*R*[*F* ^2^ > 2σ(*F* ^2^)] = 0.048
*wR*(*F* ^2^) = 0.127
*S* = 1.06
3490 reflections
271 parameters
H-atom parameters constrained
Δρ_max_ = 0.25 e Å^−3^
Δρ_min_ = −0.32 e Å^−3^

### 

Data collection: *COLLECT* (Nonius, 1998 ▶); cell refinement: *DENZO-SMN* (Otwinowski & Minor, 1997 ▶); data reduction: *DENZO*/*SCALEPACK* (Otwinowski & Minor, 1997 ▶); program(s) used to solve structure: *SHELXS86* (Sheldrick, 2008 ▶); program(s) used to refine structure: *SHELXL97* (Sheldrick, 2008 ▶); molecular graphics: *SHELXTL* (Sheldrick, 2008 ▶); software used to prepare material for publication: *SHELXTL* and *publCIF* (Westrip, 2010 ▶).

## Supplementary Material

Crystal structure: contains datablock(s) I, global. DOI: 10.1107/S1600536812032679/zl2496sup1.cif

Structure factors: contains datablock(s) I. DOI: 10.1107/S1600536812032679/zl2496Isup2.hkl

Additional supplementary materials:  crystallographic information; 3D view; checkCIF report

## Figures and Tables

**Table:** 

Mg1—O1	1.9526 (14)
Mg1—O2	1.9551 (14)
Li1—O1	1.961 (4)
Li1—O2	1.942 (4)
Li1—O3	1.988 (4)
Li1—O4	2.019 (5)
Mg1⋯Li1	2.818 (4)

**Table:** 

O1—Mg1—O1^i^	126.14 (10)
O1—Mg1—O2	87.54 (6)
O1—Mg1—O2^i^	119.89 (6)
O2—Mg1—O2^i^	119.61 (10)
O1—Li1—O2	87.69 (16)
O1—Li1—O3	125.3 (2)
O1—Li1—O4	122.7 (2)
O2—Li1—O3	120.0 (2)
O2—Li1—O4	125.3 (2)
O3—Li1—O4	80.86 (17)
Li1⋯Mg1⋯Li1^i^	174.14 (18)

Symmetry code: (i) 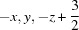.

**Fig. 1.**
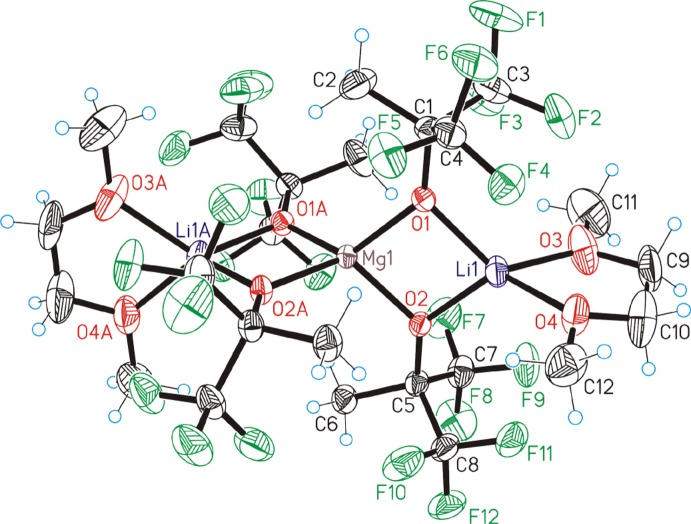
Molecular structure and labeling scheme of the title compound. Ellipsoids are drawn at the 30% probability level. Symmetry code (A): 1 - x, y, 3/2 - z.

## supplementary crystallographic information

### Comment

The synthesis of molybdenum-based Schrock-type catalysts involves the reaction of the catalyst progenitor, a Mo–trifluoromethanesulfonate compound such as Mo(N-2,6-Me_2_—C_6_H_3_)(CHCMe_2_Ph)(OTf)_2_.2DME (OTf = CF_3_SO_3_^-^), with a lithium alkoxide (Oskam *et al.*, 1993), *e.g.* LiOC(CF_3_)_2_CH_3_, to yield the corresponding Schrock catalyst Mo(N-2,6-Me_2_2-C_6_H_3_)(CHCMe_2_Ph)(OC(CF_3_)_2_CH_3_)_2_. This reaction step requires high-purity educts in which case the target compounds can be prepared in high yields. However, occasionally, lower yields are observed and could not be explained so far. Here we report on the X-ray structure of a trinuclear Li–Mg compound that forms virtually quantitatively in case the progenitor compound Mo(N-2,6-Me_2_—C_6_H_3_)(CHCMe_2_Ph)(OTf)_2_.2DME is contaminated with MgCl_2_, which is a by-product of the synthesis of this progenitor.

The structure is shown to be a trinuclear complex containing two Li cations and one central Mg cation. Selected geometry parameters are given in Table 1. The two halves of the title compound are related by a twofold axis, which passes through the central magnesium. The Mg atom is coordinated by four η^2^-bridging Li–1,1,1,3,3,3-hexafluoro-2-methylpropionate ligands that are themselves coordinated to the two Li ions. The latter have each with one 1,2-dimethoxyethane (DME) ligand a distorted tetrahedral ligand sphere. The same distorted tetrahedral ligand sphere exists for the central Mg cation.

In the crystal structure no strong intermolecular hydrogen bonds are present, only F—H distances over 2.47 Å could be observed. Therefore the displacement parameters of the CF_3_ groups and the DME ligands are comparatively large, showing a higher mobility of these atoms. An attempt to refine all non-hydrogen atoms of DME with a disordering model by splitting of the positions leads to a better *R* value, but was rejected because of too short C—O bond lengths. The distances between these split positions were only in the range of 0.28 to 0.46 Å.

### Experimental

All reactions were carried out in an MBraun glove box system (Garching, Germany) using carefully dried and deoxygenated solvents. Mo(N-2,6-Me_2_—C_6_H_3_)(CHCMe_2_Ph)(OTf)_2_.2DME (1) was prepared according to the literature (Oskam *et al.*, 1993). Briefly, ethereal solutions of 1 (1.21 g, contaminated with MgCl_2_) and LiOC(CF_3_)_2_CH_3_ (0.62 g, 3.3 mmol) were combined at -36°C and the reaction mixture was allowed to warm to room temperature. Filtration through a pad of Celite and crystallization at -36°C yielded the title complex in 10% yield.

### Refinement

All non-hydrogen atoms were refined with anisotropic displacement parameters and hydrogen atoms attached to carbon atoms were placed in calculated positions with C—H distances of 0.97 or 0.98 Å and refined with isotropic displacement parameters 1.2 or 1.5 times higher than the value of their carbon atoms.

### Crystal data

**Table:**

[Li_2_Mg(C_4_H_3_F_6_O)_4_(C_4_H_10_O_2_)_2_]	*F*(000) = 1896
*M**_r_* = 942.69	*D*_x_ = 1.584 Mg m^−^^3^
Monoclinic, *C*2/*c*	Mo *K*α radiation, λ = 0.71073 Å
Hall symbol: -C 2yc	Cell parameters from 10988 reflections
*a* = 23.8629 (4) Å	θ = 1.0–25.0°
*b* = 9.5396 (6) Å	µ = 0.20 mm^−^^1^
*c* = 18.3700 (7) Å	*T* = 233 K
β = 109.041 (2)°	Plate, colourless
*V* = 3953.0 (3) Å^3^	0.41 × 0.25 × 0.07 mm
*Z* = 4	

### Data collection

**Table:**

Nonius KappaCCD diffractometer	2603 reflections with *I* > 2σ(*I*)
Radiation source: fine-focus sealed tube	*R*_int_ = 0.031
Graphite monochromator	θ_max_ = 25.0°, θ_min_ = 2.3°
Detector resolution: 9.1 pixels mm^-1^	*h* = 0→28
φ and ω scans	*k* = −11→11
10600 measured reflections	*l* = −21→20
3490 independent reflections	

### Refinement

**Table:**

Refinement on *F*^2^	Primary atom site location: structure-invariant direct methods
Least-squares matrix: full	Secondary atom site location: difference Fourier map
*R*[*F*^2^ > 2σ(*F*^2^)] = 0.048	Hydrogen site location: inferred from neighbouring sites
*wR*(*F*^2^) = 0.127	H-atom parameters constrained
*S* = 1.06	*w* = 1/[σ^2^(*F*_o_^2^) + (0.0561*P*)^2^ + 3.6053*P*] where *P* = (*F*_o_^2^ + 2*F*_c_^2^)/3
3490 reflections	(Δ/σ)_max_ < 0.001
271 parameters	Δρ_max_ = 0.25 e Å^−^^3^
0 restraints	Δρ_min_ = −0.32 e Å^−^^3^
0 constraints	

### Special details

**Table:**

Geometry. All e.s.d.'s (except the e.s.d. in the dihedral angle between two l.s. planes) are estimated using the full covariance matrix. The cell e.s.d.'s are taken into account individually in the estimation of e.s.d.'s in distances, angles and torsion angles; correlations between e.s.d.'s in cell parameters are only used when they are defined by crystal symmetry. An approximate (isotropic) treatment of cell e.s.d.'s is used for estimating e.s.d.'s involving l.s. planes.
Refinement. Refinement of *F*^2^ against ALL reflections. The weighted *R*-factor *wR* and goodness of fit *S* are based on *F*^2^, conventional *R*-factors *R* are based on *F*, with *F* set to zero for negative *F*^2^. The threshold expression of *F*^2^ > σ(*F*^2^) is used only for calculating *R*-factors(gt) *etc*. and is not relevant to the choice of reflections for refinement. *R*-factors based on *F*^2^ are statistically about twice as large as those based on *F*, and *R*-factors based on ALL data will be even larger.

### Fractional atomic coordinates and isotropic or equivalent isotropic displacement parameters (Å^2^)

**Table:**

	*x*	*y*	*z*	*U*_iso_*/*U*_eq_	
Mg1	0.0000	0.21820 (10)	0.7500	0.0304 (2)	
Li1	0.12432 (17)	0.2031 (5)	0.8154 (2)	0.0488 (10)	
O1	0.06054 (6)	0.31089 (15)	0.83443 (8)	0.0376 (4)	
O2	0.06414 (6)	0.11513 (16)	0.72966 (8)	0.0380 (4)	
O3	0.19633 (10)	0.2846 (3)	0.80007 (14)	0.0967 (8)	
O4	0.18689 (8)	0.1002 (3)	0.89956 (13)	0.0788 (6)	
C1	0.06148 (11)	0.4201 (2)	0.88423 (13)	0.0452 (6)	
C2	0.00224 (14)	0.4955 (3)	0.86486 (17)	0.0737 (9)	
H2A	−0.0060	0.5439	0.8160	0.111*	
H2B	0.0038	0.5629	0.9050	0.111*	
H2C	−0.0289	0.4278	0.8612	0.111*	
C3	0.10945 (15)	0.5255 (3)	0.88057 (17)	0.0667 (8)	
C4	0.07842 (12)	0.3634 (3)	0.96686 (13)	0.0534 (7)	
C5	0.06465 (10)	0.0184 (3)	0.67423 (12)	0.0421 (6)	
C6	0.00260 (11)	−0.0155 (3)	0.61935 (14)	0.0542 (7)	
H6A	0.0054	−0.0857	0.5825	0.081*	
H6B	−0.0153	0.0689	0.5922	0.081*	
H6C	−0.0216	−0.0512	0.6486	0.081*	
C7	0.10204 (12)	0.0747 (3)	0.62675 (15)	0.0596 (7)	
C8	0.09244 (12)	−0.1182 (3)	0.71356 (15)	0.0555 (7)	
C9	0.24947 (14)	0.2386 (5)	0.8556 (3)	0.1029 (13)	
H9A	0.2652	0.3139	0.8929	0.124*	
H9B	0.2788	0.2190	0.8300	0.124*	
C10	0.24142 (16)	0.1150 (7)	0.8958 (3)	0.1183 (16)	
H10A	0.2508	0.0326	0.8700	0.142*	
H10B	0.2697	0.1175	0.9483	0.142*	
C11	0.2021 (2)	0.3924 (5)	0.7506 (3)	0.1287 (17)	
H11A	0.1634	0.4151	0.7144	0.193*	
H11B	0.2280	0.3614	0.7226	0.193*	
H11C	0.2189	0.4749	0.7807	0.193*	
C12	0.17925 (17)	−0.0164 (4)	0.9429 (2)	0.0917 (11)	
H12A	0.1382	−0.0216	0.9409	0.138*	
H12B	0.2045	−0.0059	0.9960	0.138*	
H12C	0.1898	−0.1015	0.9216	0.138*	
F1	0.10808 (12)	0.6453 (2)	0.91690 (13)	0.1145 (8)	
F2	0.16449 (8)	0.4746 (2)	0.91064 (10)	0.0845 (6)	
F3	0.10296 (9)	0.55831 (18)	0.80778 (10)	0.0865 (6)	
F4	0.12382 (7)	0.27533 (18)	0.98428 (8)	0.0694 (5)	
F5	0.03358 (8)	0.2936 (2)	0.97813 (9)	0.0771 (5)	
F6	0.09337 (9)	0.4647 (2)	1.02026 (9)	0.0849 (6)	
F7	0.08268 (9)	0.1986 (2)	0.59720 (12)	0.0964 (7)	
F8	0.10134 (10)	−0.0077 (3)	0.56813 (11)	0.1073 (8)	
F9	0.15870 (7)	0.0932 (2)	0.66710 (11)	0.0895 (6)	
F10	0.05962 (9)	−0.17323 (19)	0.75248 (12)	0.0885 (6)	
F11	0.14670 (8)	−0.10129 (19)	0.76398 (10)	0.0828 (6)	
F12	0.09778 (8)	−0.21737 (18)	0.66513 (11)	0.0857 (6)	

### Atomic displacement parameters (Å^2^)

**Table:**

	*U*^11^	*U*^22^	*U*^33^	*U*^12^	*U*^13^	*U*^23^
Mg1	0.0262 (5)	0.0337 (5)	0.0298 (5)	0.000	0.0072 (4)	0.000
Li1	0.033 (2)	0.062 (3)	0.048 (2)	−0.0033 (18)	0.0095 (17)	−0.0061 (19)
O1	0.0339 (8)	0.0390 (9)	0.0359 (8)	−0.0013 (6)	0.0058 (6)	−0.0057 (6)
O2	0.0297 (8)	0.0446 (9)	0.0388 (8)	0.0019 (6)	0.0099 (6)	−0.0085 (7)
O3	0.0595 (15)	0.147 (2)	0.0865 (16)	−0.0383 (14)	0.0279 (12)	0.0027 (15)
O4	0.0418 (12)	0.0969 (17)	0.0879 (15)	0.0052 (10)	0.0077 (10)	0.0001 (13)
C1	0.0489 (14)	0.0404 (13)	0.0406 (12)	0.0023 (11)	0.0066 (10)	−0.0092 (11)
C2	0.073 (2)	0.075 (2)	0.0640 (18)	0.0296 (16)	0.0095 (15)	−0.0161 (15)
C3	0.089 (2)	0.0455 (17)	0.0557 (17)	−0.0132 (15)	0.0098 (15)	−0.0078 (13)
C4	0.0565 (17)	0.0617 (17)	0.0384 (13)	−0.0034 (14)	0.0103 (11)	−0.0119 (12)
C5	0.0365 (13)	0.0509 (14)	0.0392 (12)	0.0032 (10)	0.0129 (9)	−0.0094 (11)
C6	0.0432 (15)	0.0642 (17)	0.0494 (14)	0.0004 (12)	0.0071 (11)	−0.0146 (13)
C7	0.0499 (17)	0.082 (2)	0.0506 (15)	0.0043 (14)	0.0218 (12)	−0.0077 (15)
C8	0.0495 (16)	0.0560 (17)	0.0580 (16)	0.0131 (12)	0.0132 (13)	−0.0126 (13)
C9	0.0324 (18)	0.139 (4)	0.130 (3)	−0.018 (2)	0.0162 (19)	−0.029 (3)
C10	0.046 (2)	0.188 (5)	0.113 (3)	0.004 (3)	0.015 (2)	0.012 (3)
C11	0.125 (4)	0.147 (4)	0.132 (4)	−0.052 (3)	0.067 (3)	0.006 (3)
C12	0.092 (3)	0.098 (3)	0.074 (2)	0.017 (2)	0.0123 (19)	0.013 (2)
F1	0.169 (2)	0.0519 (11)	0.1148 (16)	−0.0323 (13)	0.0355 (15)	−0.0310 (11)
F2	0.0605 (12)	0.1003 (14)	0.0786 (11)	−0.0306 (10)	0.0034 (9)	0.0063 (10)
F3	0.1160 (16)	0.0649 (11)	0.0704 (11)	−0.0227 (10)	0.0192 (10)	0.0165 (9)
F4	0.0675 (11)	0.0814 (11)	0.0496 (9)	0.0126 (9)	0.0058 (7)	0.0119 (8)
F5	0.0748 (12)	0.1057 (14)	0.0553 (9)	−0.0207 (10)	0.0273 (8)	−0.0062 (9)
F6	0.1072 (15)	0.0909 (13)	0.0485 (9)	−0.0129 (11)	0.0143 (9)	−0.0317 (9)
F7	0.0931 (14)	0.1067 (16)	0.1117 (15)	0.0178 (12)	0.0638 (12)	0.0447 (13)
F8	0.1226 (17)	0.1462 (19)	0.0784 (13)	−0.0195 (14)	0.0674 (13)	−0.0434 (13)
F9	0.0449 (10)	0.1405 (18)	0.0897 (13)	−0.0109 (10)	0.0310 (9)	−0.0069 (12)
F10	0.0994 (14)	0.0670 (11)	0.1148 (15)	0.0272 (10)	0.0566 (12)	0.0320 (10)
F11	0.0639 (11)	0.0816 (12)	0.0795 (11)	0.0237 (9)	−0.0085 (9)	−0.0071 (9)
F12	0.0897 (13)	0.0682 (11)	0.0926 (13)	0.0250 (9)	0.0208 (10)	−0.0300 (10)

### Geometric parameters (Å, º)

**Table:**

Mg1—O1	1.9526 (14)	C4—F4	1.325 (3)
Mg1—O1^i^	1.9526 (14)	C4—F5	1.333 (3)
Mg1—O2	1.9551 (14)	C4—F6	1.340 (3)
Mg1—O2^i^	1.9551 (14)	C5—C6	1.530 (3)
Mg1—Li1	2.818 (4)	C5—C8	1.532 (4)
Mg1—Li1^i^	2.818 (4)	C5—C7	1.534 (4)
Li1—O1	1.961 (4)	C6—H6A	0.9700
Li1—O2	1.942 (4)	C6—H6B	0.9700
Li1—O3	1.988 (4)	C6—H6C	0.9700
Li1—O4	2.019 (5)	C7—F7	1.319 (3)
Mg1—Li1	2.818 (4)	C7—F9	1.325 (3)
O1—C1	1.382 (3)	C7—F8	1.329 (3)
O2—C5	1.377 (3)	C8—F10	1.329 (3)
O3—C11	1.409 (5)	C8—F12	1.333 (3)
O3—C9	1.413 (5)	C8—F11	1.333 (3)
O4—C10	1.333 (4)	C9—C10	1.437 (7)
O4—C12	1.414 (4)	C9—H9A	0.9800
C1—C2	1.522 (4)	C9—H9B	0.9800
C1—C4	1.536 (3)	C10—H10A	0.9800
C1—C3	1.541 (4)	C10—H10B	0.9800
C2—H2A	0.9700	C11—H11A	0.9700
C2—H2B	0.9700	C11—H11B	0.9700
C2—H2C	0.9700	C11—H11C	0.9700
C3—F1	1.329 (3)	C12—H12A	0.9700
C3—F3	1.332 (3)	C12—H12B	0.9700
C3—F2	1.339 (4)	C12—H12C	0.9700
			
O1—Mg1—O1^i^	126.14 (10)	F4—C4—F5	106.2 (2)
O1—Mg1—O2	87.54 (6)	F4—C4—F6	106.2 (2)
O1^i^—Mg1—O2	119.89 (6)	F5—C4—F6	106.4 (2)
O1—Mg1—O2^i^	119.89 (6)	F4—C4—C1	113.1 (2)
O1^i^—Mg1—O2^i^	87.54 (6)	F5—C4—C1	111.3 (2)
O2—Mg1—O2^i^	119.61 (10)	F6—C4—C1	113.0 (2)
O1—Mg1—Li1	44.06 (9)	O2—C5—C6	112.89 (18)
O1^i^—Mg1—Li1	139.90 (10)	O2—C5—C8	109.16 (18)
O2—Mg1—Li1	43.50 (9)	C6—C5—C8	107.9 (2)
O2^i^—Mg1—Li1	132.37 (10)	O2—C5—C7	109.4 (2)
O1—Mg1—Li1^i^	139.90 (10)	C6—C5—C7	108.5 (2)
O1^i^—Mg1—Li1^i^	44.06 (9)	C8—C5—C7	108.9 (2)
O2—Mg1—Li1^i^	132.37 (10)	C5—C6—H6A	109.5
O2^i^—Mg1—Li1^i^	43.50 (9)	C5—C6—H6B	109.5
O1—Li1—O2	87.69 (16)	H6A—C6—H6B	109.5
O1—Li1—O3	125.3 (2)	C5—C6—H6C	109.5
O1—Li1—O4	122.7 (2)	H6A—C6—H6C	109.5
O2—Li1—O3	120.0 (2)	H6B—C6—H6C	109.5
O2—Li1—O4	125.3 (2)	F7—C7—F9	105.5 (3)
O3—Li1—O4	80.86 (17)	F7—C7—F8	106.6 (2)
Li1—Mg1—Li1^i^	174.14 (18)	F9—C7—F8	105.8 (2)
O2—Li1—Mg1	43.88 (9)	F7—C7—C5	110.9 (2)
O1—Li1—Mg1	43.83 (9)	F9—C7—C5	113.9 (2)
O3—Li1—Mg1	139.3 (2)	F8—C7—C5	113.4 (2)
O4—Li1—Mg1	139.8 (2)	F10—C8—F12	106.3 (2)
C1—O1—Mg1	135.67 (14)	F10—C8—F11	106.5 (2)
C1—O1—Li1	131.90 (18)	F12—C8—F11	105.5 (2)
Mg1—O1—Li1	92.11 (13)	F10—C8—C5	110.5 (2)
C5—O2—Li1	134.95 (17)	F12—C8—C5	114.2 (2)
C5—O2—Mg1	132.43 (13)	F11—C8—C5	113.4 (2)
Li1—O2—Mg1	92.62 (13)	O3—C9—C10	112.7 (3)
C11—O3—C9	116.0 (3)	O3—C9—H9A	109.0
C11—O3—Li1	130.4 (3)	C10—C9—H9A	109.0
C9—O3—Li1	113.0 (3)	O3—C9—H9B	109.0
C10—O4—C12	114.8 (3)	C10—C9—H9B	109.0
C10—O4—Li1	113.5 (3)	H9A—C9—H9B	107.8
C12—O4—Li1	127.9 (2)	O4—C10—C9	114.2 (4)
O1—C1—C2	112.9 (2)	O4—C10—H10A	108.7
O1—C1—C4	109.35 (19)	C9—C10—H10A	108.7
C2—C1—C4	108.8 (2)	O4—C10—H10B	108.7
O1—C1—C3	108.3 (2)	C9—C10—H10B	108.7
C2—C1—C3	109.1 (2)	H10A—C10—H10B	107.6
C4—C1—C3	108.2 (2)	O3—C11—H11A	109.5
C1—C2—H2A	109.5	O3—C11—H11B	109.5
C1—C2—H2B	109.5	H11A—C11—H11B	109.5
H2A—C2—H2B	109.5	O3—C11—H11C	109.5
C1—C2—H2C	109.5	H11A—C11—H11C	109.5
H2A—C2—H2C	109.5	H11B—C11—H11C	109.5
H2B—C2—H2C	109.5	O4—C12—H12A	109.5
F1—C3—F3	106.7 (2)	O4—C12—H12B	109.5
F1—C3—F2	106.6 (2)	H12A—C12—H12B	109.5
F3—C3—F2	106.1 (3)	O4—C12—H12C	109.5
F1—C3—C1	113.5 (3)	H12A—C12—H12C	109.5
F3—C3—C1	110.6 (2)	H12B—C12—H12C	109.5
F2—C3—C1	112.8 (2)		
			
O1—Mg1—Li1—O2	−177.8 (2)	O2—Li1—O4—C12	−47.5 (4)
O1^i^—Mg1—Li1—O2	−82.68 (18)	O1—Li1—O4—C12	65.9 (4)
O2^i^—Mg1—Li1—O2	90.60 (17)	O3—Li1—O4—C12	−167.8 (3)
O1^i^—Mg1—Li1—O1	95.15 (18)	Mg1—Li1—O4—C12	10.5 (5)
O2—Mg1—Li1—O1	177.8 (2)	Mg1—O1—C1—C2	−6.4 (3)
O2^i^—Mg1—Li1—O1	−91.57 (15)	Li1—O1—C1—C2	165.2 (2)
O1—Mg1—Li1—O3	−93.8 (3)	Mg1—O1—C1—C4	114.8 (2)
O1^i^—Mg1—Li1—O3	1.3 (4)	Li1—O1—C1—C4	−73.6 (3)
O2—Mg1—Li1—O3	84.0 (3)	Mg1—O1—C1—C3	−127.4 (2)
O2^i^—Mg1—Li1—O3	174.6 (2)	Li1—O1—C1—C3	44.2 (3)
O1—Mg1—Li1—O4	88.7 (3)	O1—C1—C3—F1	170.0 (2)
O1^i^—Mg1—Li1—O4	−176.1 (2)	C2—C1—C3—F1	46.8 (3)
O2—Mg1—Li1—O4	−93.5 (3)	C4—C1—C3—F1	−71.5 (3)
O2^i^—Mg1—Li1—O4	−2.9 (4)	O1—C1—C3—F3	50.1 (3)
O1^i^—Mg1—O1—C1	46.36 (19)	C2—C1—C3—F3	−73.1 (3)
O2—Mg1—O1—C1	172.3 (2)	C4—C1—C3—F3	168.6 (2)
O2^i^—Mg1—O1—C1	−64.7 (2)	O1—C1—C3—F2	−68.5 (3)
Li1—Mg1—O1—C1	173.8 (3)	C2—C1—C3—F2	168.2 (2)
Li1^i^—Mg1—O1—C1	−12.7 (3)	C4—C1—C3—F2	50.0 (3)
O1^i^—Mg1—O1—Li1	−127.40 (14)	O1—C1—C4—F4	45.1 (3)
O2—Mg1—O1—Li1	−1.49 (14)	C2—C1—C4—F4	168.8 (2)
O2^i^—Mg1—O1—Li1	121.59 (14)	C3—C1—C4—F4	−72.8 (3)
Li1^i^—Mg1—O1—Li1	173.5 (2)	O1—C1—C4—F5	−74.5 (3)
O2—Li1—O1—C1	−172.63 (19)	C2—C1—C4—F5	49.2 (3)
O3—Li1—O1—C1	−46.9 (4)	C3—C1—C4—F5	167.7 (2)
O4—Li1—O1—C1	56.0 (4)	O1—C1—C4—F6	165.8 (2)
Mg1—Li1—O1—C1	−174.1 (2)	C2—C1—C4—F6	−70.5 (3)
O2—Li1—O1—Mg1	1.51 (14)	C3—C1—C4—F6	48.0 (3)
O3—Li1—O1—Mg1	127.2 (2)	Li1—O2—C5—C6	179.6 (2)
O4—Li1—O1—Mg1	−129.9 (2)	Mg1—O2—C5—C6	−1.2 (3)
O1—Li1—O2—C5	177.9 (2)	Li1—O2—C5—C8	59.6 (3)
O3—Li1—O2—C5	47.8 (4)	Mg1—O2—C5—C8	−121.24 (19)
O4—Li1—O2—C5	−52.7 (4)	Li1—O2—C5—C7	−59.5 (3)
Mg1—Li1—O2—C5	179.4 (3)	Mg1—O2—C5—C7	119.70 (19)
O1—Li1—O2—Mg1	−1.50 (14)	O2—C5—C7—F7	−55.3 (3)
O3—Li1—O2—Mg1	−131.6 (2)	C6—C5—C7—F7	68.2 (3)
O4—Li1—O2—Mg1	127.9 (2)	C8—C5—C7—F7	−174.6 (2)
O1—Mg1—O2—C5	−177.90 (19)	O2—C5—C7—F9	63.5 (3)
O1^i^—Mg1—O2—C5	−46.9 (2)	C6—C5—C7—F9	−172.9 (2)
O2^i^—Mg1—O2—C5	58.78 (18)	C8—C5—C7—F9	−55.7 (3)
Li1—Mg1—O2—C5	−179.4 (3)	O2—C5—C7—F8	−175.3 (2)
Li1^i^—Mg1—O2—C5	6.4 (2)	C6—C5—C7—F8	−51.7 (3)
O1—Mg1—O2—Li1	1.51 (14)	C8—C5—C7—F8	65.5 (3)
O1^i^—Mg1—O2—Li1	132.53 (14)	O2—C5—C8—F10	63.4 (3)
O2^i^—Mg1—O2—Li1	−121.82 (14)	C6—C5—C8—F10	−59.7 (3)
Li1^i^—Mg1—O2—Li1	−174.16 (18)	C7—C5—C8—F10	−177.2 (2)
O2—Li1—O3—C11	60.1 (5)	O2—C5—C8—F12	−176.9 (2)
O1—Li1—O3—C11	−50.4 (5)	C6—C5—C8—F12	60.0 (3)
O4—Li1—O3—C11	−174.3 (4)	C7—C5—C8—F12	−57.5 (3)
Mg1—Li1—O3—C11	7.4 (5)	O2—C5—C8—F11	−56.0 (3)
O2—Li1—O3—C9	−129.6 (3)	C6—C5—C8—F11	−179.1 (2)
O1—Li1—O3—C9	119.9 (3)	C7—C5—C8—F11	63.4 (3)
O4—Li1—O3—C9	−4.0 (3)	C11—O3—C9—C10	−171.0 (4)
Mg1—Li1—O3—C9	177.7 (3)	Li1—O3—C9—C10	17.2 (5)
O2—Li1—O4—C10	109.4 (4)	C12—O4—C10—C9	−176.4 (4)
O1—Li1—O4—C10	−137.2 (3)	Li1—O4—C10—C9	23.6 (5)
O3—Li1—O4—C10	−10.9 (3)	O3—C9—C10—O4	−27.0 (6)
Mg1—Li1—O4—C10	167.4 (4)		

Symmetry code: (i) −*x*, *y*, −*z*+3/2.
